# Seasonal Dynamics of Water Use Strategy of Two *Salix* Shrubs in Alpine Sandy Land, Tibetan Plateau

**DOI:** 10.1371/journal.pone.0156586

**Published:** 2016-05-31

**Authors:** Yajuan Zhu, Guojie Wang, Renqiang Li

**Affiliations:** 1 Institute of Desertification Studies, Chinese Academy of Forestry, Beijing, China; 2 School of Environmental Science and Engineering, Nanjing University of Information Science & Technology, Nanjing, Jiangsu, China; 3 Oregon State University Agriculture Program at Eastern Oregon University, Oregon State University, La Grande, OR, United States of America; 4 Key Laboratory of Ecosystem Network Observation and Modeling, Institute of Geographic Sciences and Natural Resources Research, Chinese Academy of Sciences, Beijing, China; Estación Experimental del Zaidín (CSIC), SPAIN

## Abstract

Water is a limiting factor for plant growth and vegetation dynamics in alpine sandy land of the Tibetan Plateau, especially with the increasing frequency of extreme precipitation events and drought caused by climate change. Therefore, a relatively stable water source from either deeper soil profiles or ground water is necessary for plant growth. Understanding the water use strategy of dominant species in the alpine sandy land ecosystem is important for vegetative rehabilitation and ecological restoration. The stable isotope methodology of δD, δ^18^O, and δ^13^C was used to determine main water source and long-term water use efficiency of *Salix psammophila* and *S*. *cheilophila*, two dominant shrubs on interdune of alpine sandy land in northeastern Tibetan Plateau. The root systems of two *Salix* shrubs were investigated to determine their distribution pattern. The results showed that *S*. *psammophila* and *S*. *cheilophila* absorbed soil water at different soil depths or ground water in different seasons, depending on water availability and water use strategy. *Salix psammophila* used ground water during the growing season and relied on shallow soil water recharged by rain in summer. *Salix cheilophila* used ground water in spring and summer, but relied on shallow soil water recharged by rain in spring and deep soil water recharged by ground water in fall. The two shrubs had dimorphic root systems, which is coincident with their water use strategy. Higher biomass of fine roots in *S*. *psammophila* and longer fine roots in *S*. *cheilophila* facilitated to absorb water in deeper soil layers. The long-term water use efficiency of two *Salix* shrubs increased during the dry season in spring. The long-term water use efficiency was higher in *S*. *psammophila* than in *S*. *cheilophila*, as the former species is better adapted to semiarid climate of alpine sandy land.

## Introduction

The alpine sandy land of Gonghe Basin is located in the northeastern Tibetan Plateau, which is an ecotone from semi-arid steppe to arid desert steppe and with the altitude ranging from 2600 m to 3400 m. It is one of the most severely desertified area in Qinghai Province [1]; desertification is mainly caused by climate change [2] and human activities [3]. In order to control and prevent land desertification, large area trees and shrubs were planted to form a shelterbelt system to protect farms, villages, and roads. Thus, trees from the genus *Populus* were planted inside oasis, *Salix* shrubs were planted on interdune, and shrubs from the genus *Caragana* were planted on sand dunes. This approach was successful as the shelterbelt system decreased sand storms and improved microclimate [4].

In arid and semi-arid ecosystems, which have low and unpredictable precipitation and high evapotranspiration potential, water is a limiting factor for plant growth and it directly affects community structure and ecosystem functioning [5–7]. The climate change model predicted that the frequency of extreme precipitation events and drought in these ecosystems will increase with global warming [8]. Therefore, the ability to use rainwater in spring and summer is important for plant phenology and growth [9–10]. In addition, the stable water source from either deep soil profiles or ground water is necessary for plant growth especially under drought conditions [11].

Stable isotope technology has been widely applied to study water use strategy of plant species in arid ecosystems, including main water source and water use efficiency. There is no stable isotope fractionation during water uptake by root system or water transpiration flux in the xylem of most plant species [5]. Therefore, the main water source for a plant can be examined by comparing the stable hydrogen or oxygen value of xylem water with that of the potential water source, including rain, snow, river, soil water, or ground water [5,11]. Furthermore, the leaf carbon isotope value of C_3_ plants is positively related to their long-term water use efficiency [12]. The δ^13^C value drops from spring to fall during the growing season, and it increases with the increase of plant age [13,14] and under drought conditions [11]. Previous studies with stable isotopes of hydrogen and oxygen indicated that plant species in arid and semi-arid regions used different source water. Many trees, shrubs, and perennial grasses mainly used shallow or middle soil water recharged by rain [12, 15–21]. Some shrubs mainly used deep soil water recharged by snow [22] or deep soil water recharged by large amount of rainfall [23], and still other shrubs and trees utilized ground water [14,21,23,24–28].

The differences in water use strategy of different species are achieved by their specific root systems [22]. Plants such as shrubs in arid and semi-arid regions develop extensive root system to absorb soil water. Thus, shrubs develop either vertical root systems to access deep soil water [13,29,30] or extensive horizontal root systems to absorb shallow soil water [30,31]. A few shrubs possess dimorphic root systems that use both shallow and deep soil water, or even ground water [21,31,32].

Our earlier research [13] indicated that *Caragana intermedia*, a dominant shrub on sand dunes of alpine sandy land ecosystem in northeastern Tibetan Plateau, changes its water use strategy as an adaptation to semi-arid environment, i.e., the depth of soil water use and the long-term water use efficiency increases with the species plantation age. However, water use strategy of dominant species on interdune in this fragile ecosystem is yet to be elucidated, which is crucial for combating desertification and ecological restoration of degraded grasslands. Therefore, the objective of this study was to quantify water use strategy of two dominant shrubs, *Salix psammophila* and *Salix cheilophila*, using stable isotope methodology and determine relation of their root systems and soil water availability on interdune of alpine sandy land in Tibetan Plateau.

## Materials and Methods

### Study Site

The field study was conducted at the Qinghai Gonghe Desert Ecosystem Research Station, located in Gonghe Basin, northeastern Tibetan Plateau (36°16ʹN, 100°16ʹE, and altitude 2874 m). The station was co-created in 2007 by Chinese Academy of Forestry and Qinghai Desert Control Station. The mean annual air temperature is only 2.4°C, with 91 frost-free days. The mean air temperature is 12.6°C in the growing season (from May to September). The mean annual precipitation is 246.3 mm, rain mainly concentrated from July to September. The major vegetation types are temperate grassland dominated by *Achnatherum splendens* and shrub land dominated by *Artemisia ordosica*, *Nitraria tangutorum* and *Caragana tibetica*. Shelterbelts were formed by trees and shrubs to protect the oasis—*Populus cathayana* were planted in farms and villages, *Salix psammophila*, *Salix cheilophila*, and *Hippophae rhamnoides* on interdune and *Caragana intermedia*, *Caragana korshinskii* and *Caragana microphylla* on sand dunes. Soil on interdune is sandy loam with 20.28% clay and 79.72% sand. The detailed description of the study area can be found in our earlier study [13].

### Plant Species

*Salix psammophila* (sandy willow) is a shrub 2–4 m high, inhabiting moving or semi-fixed sand dunes and interdune. The species is fast growing, tolerant to drought and sand burial, and a good sand-fixing plant [33]. *Salix cheilophila* (black willow) is a small tree or big shrub up to 5.4 m tall, inhabiting valley slopes, valley bottoms, and river banks. The species is a mesophyte and hygrophyte and often used as a sand-fixing or riparian plant [34]. Both species were planted by cuttage in 1986 in the study area. The plantation of two *Salix* is mixed forest with two types of belts. The planting density was 1 m × 2 m for two *Salix* shrubs. Five rows of *S*. *psammophila* formed a belt, which is adjacent to another two belts formed by three rows of *S*. *cheilophila*. The distance between two belts was 5 m. Four plots of 5 m × 5 m were set separately in four different *S*. *psammophila* and *S*. *cheilophila* belts, for field sampling purposes. The mean heights of *S*. *psammophila* and *S*. *cheilophila* were 2.81 ± 0.37 m and 3.69 ± 0.23 m, respectively.

### Precipitation in the Growing Season of 2014

**Fig 1:**
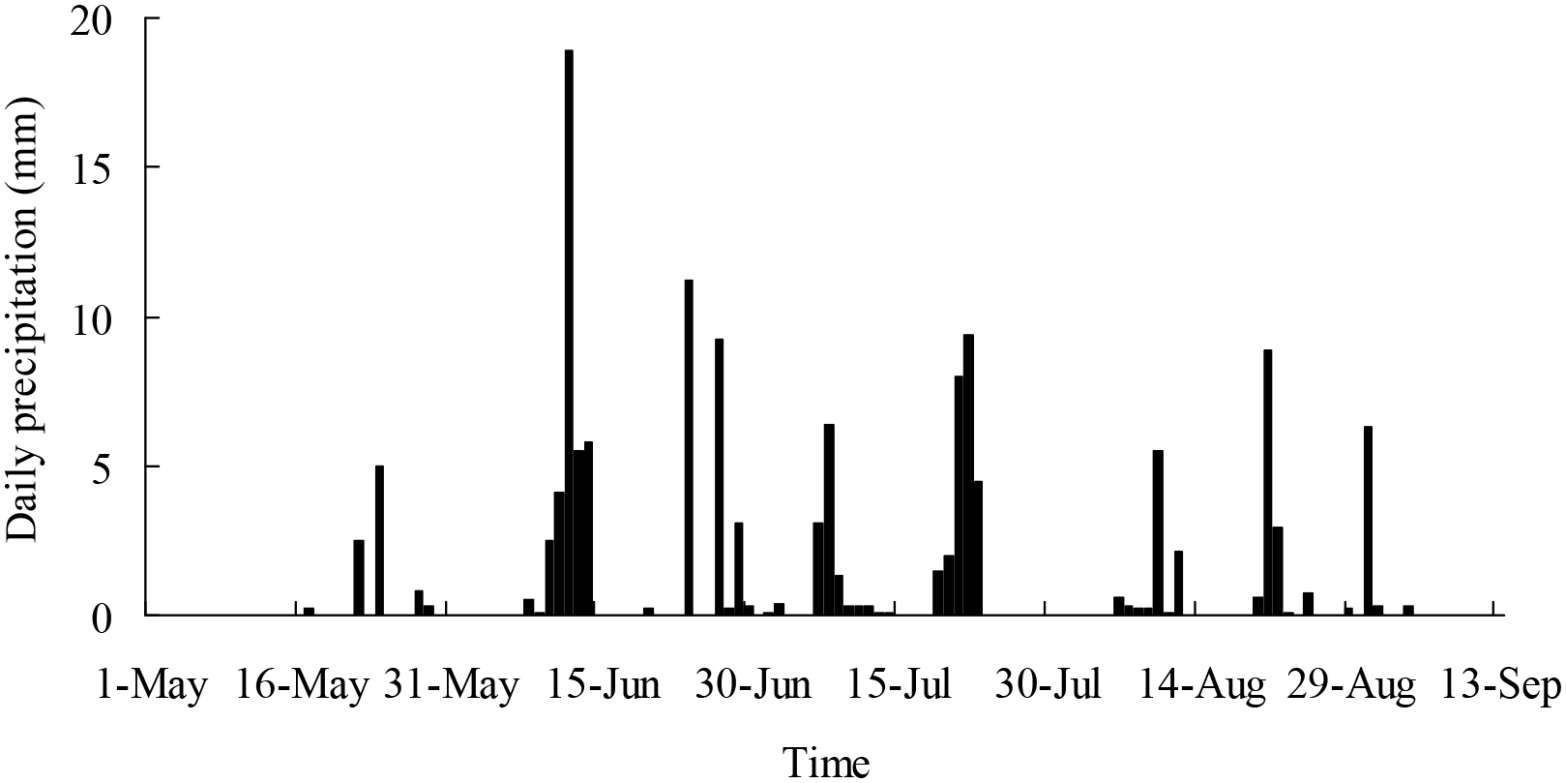
Daily precipitation at Gonghe Station from May 1 to September 13, 2014.

The total precipitation at the study site was 137.5 mm from May 1 to September 13, 2014. The monthly precipitation in May, June, July, and August was 8.8, 61.6, 37.8, and 28.7 mm, respectively. The maximal daily precipitation (18.9 mm) occurred on June 12 (Fig 1).

### Field Sampling and Measurements

The study site is national land of the Qinghai Desert Control Station, which granted permission to conduct the study on this site. None of the field experiments involved endangered or protected species. Soil samples were collected in spring (May 26), summer (July 17), and fall (September 11) in 2014 by soil auger with 6.99 cm diameter (AMS Inc., American Falls, ID, USA) from the middle point of two adjacent rows in the four plots with the two *Salix* species. The depth of soil sampling was determined by rooting depth of the two shrubs were 10, 25, 50, 75, 100, and 150 cm for *S*. *psammophila* and 10, 25, 50, 100, 150, and 200 cm for *S*. *cheilophila*. Soil samples were placed into 8-mL glass vials (National Scientific Company, Rockwood, TN, USA), sealed with Parafilm^®^ (Alcan Packaging, Chicago, IL, USA), and stored in a 16-L medical cool box at < 15°C. Four replicates of each soil sample were placed in aluminum can and used to measure soil water content. The wet soil sample in each aluminum can was weighed on an electronic balance (± 0.01 g), dried at 105°C for 24 h, and weighed again; the soil water content (g kg^-1^) was calculated as the loss of water.

Twigs of two *Salix* shrubs were sampled on May 26, July 17 and September 11, 2014. Lignified, two years old twigs (5 cm long, 3–5 mm diameter) were collected with scissor from the sunny side of four shrubs adjacent to the soil sampling spot in the four plots with the two *Salix* species. The bark was removed by scissor and the xylem was placed in 8 mL glass vials, sealed with Parafilm^®^, and stored in a medical box at < 15°C. Rain water was collected on rainy days at Gonghe Station before soil sampling. Well water was collected from a 5 m deep well located 1 km from the study plot on May 26, July 17, and September 11 and used as a surrogate for ground water. Three replicates of water samples were placed into 8 mL glass vials, sealed with Parafilm^®^, and stored in a medical box at < 15°C. Water in soil and xylem samples was vacuum-extracted with LI-2000 plant and soil water vacuum extract system (LICA United Technology Limited, Beijing, China). The δD and δ^18^O value of soil water, xylem water, along with the collected rain and well water was measured with a Flash 2000 HT elemental analyzer and a Finnigan MAT 253 mass spectrometer (Thermo Finnigan GmbH, Bremen, Germany) in the Stable Isotope Ecology Laboratory, Tsinghua University. The accuracy of δD and δ^18^O measurement is ± 1‰ and ± 0.2‰, respectively. A water sample was measured for three times and the mean value of three measurements was presented.

Leaves of two *Salix* shrubs were sampled on May 26, July 17 and September 11, 2014. Four replicates of mature leaf samples were collected randomly from the sunny side of the four shrubs which twigs were sampled, placed in paper bags, and dried at 75°C for 48 h. Dry leaves were pulverized and passed through an 80 mesh sieve. The δ^13^C value of leaves was measured with the Flash 2000 HT elemental analyzer and the Finnigan MAT 253 mass spectrometer in the same laboratory for δD and δ^18^O value. The accuracy of δ^13^C measurement is ± 0.1‰. A leaf sample was measured for three times and the mean value of three measurements was presented. The long-term water use efficiency of two *Salix* shrubs is calculated by their leaf δ^13^C value [35].

The root survey was conducted from July 15 to July 17, 2014. Three replicates of each soil profile (50 cm × 50 cm) were dug out by shovel for two *Salix* shrubs. Soil samples were collected at every 10 cm depth and passed through an 18 mesh sieve to collect roots until fine root is invisible. The maximal depth of surveyed roots was 110 cm for *S*. *psammophila* and 120 cm for *S*. *cheilophila*. The recovered roots were divided into three categories according to the diameter, which was measured with vernier caliper (± 0.02 mm): prop roots > 5 mm, medium roots 1–5 mm, and fine roots < 1 mm diameter. The length of different types of roots was measured with a ruler (± 1 mm) and transformed into length density (m m^-3^). After root diameter and length were measured, the roots were placed into paper bags, dried at 75°C for 48 h, and weighed on an electronic balance (± 0.01 g). Root dry mass (g) was transformed into root density (g m^-3^).

### Data Analysis

Soil water content, root length and mass density, water use efficiency and value of δD, δ^18^O, and δ^13^C were expressed as mean ± SE. One-way analysis of variance was used to compare the effects of soil depth on soil water content, root length or mass density, and the effects of month on δ^13^C value and water use efficiency by SPSS 19.0 (IBM Corp., Armonk, NY, USA). If the effects were statistically significant (*P* < 0.05), Duncan’s multiple-range test at the significance of 0.05 was used to compare the difference of soil water content, root length or mass density between different soil depths and the difference of leaf δ^13^C value and water use efficiency between different months. Water use ratio of different sources was analyzed by IsoSource 1.3.1 with 1% source increment and 0.1% mass balance tolerance [36] (download from https://www.epa.gov/eco-research/stable-isotope-mixing-models-estimating-source-proportions). IsoSource is a stable isotope mixing model for portioning an excess number of sources, such as water sources for plant uptake only if there is significant difference in different source water. It provides the distribution of source proportions which are consistent with isotopic mass balance. The results of water use ratio to different sources were expressed as mean ± SD.

## Results

### Soil Water Content in *Salix* Shelterbelts

**Fig 2:**
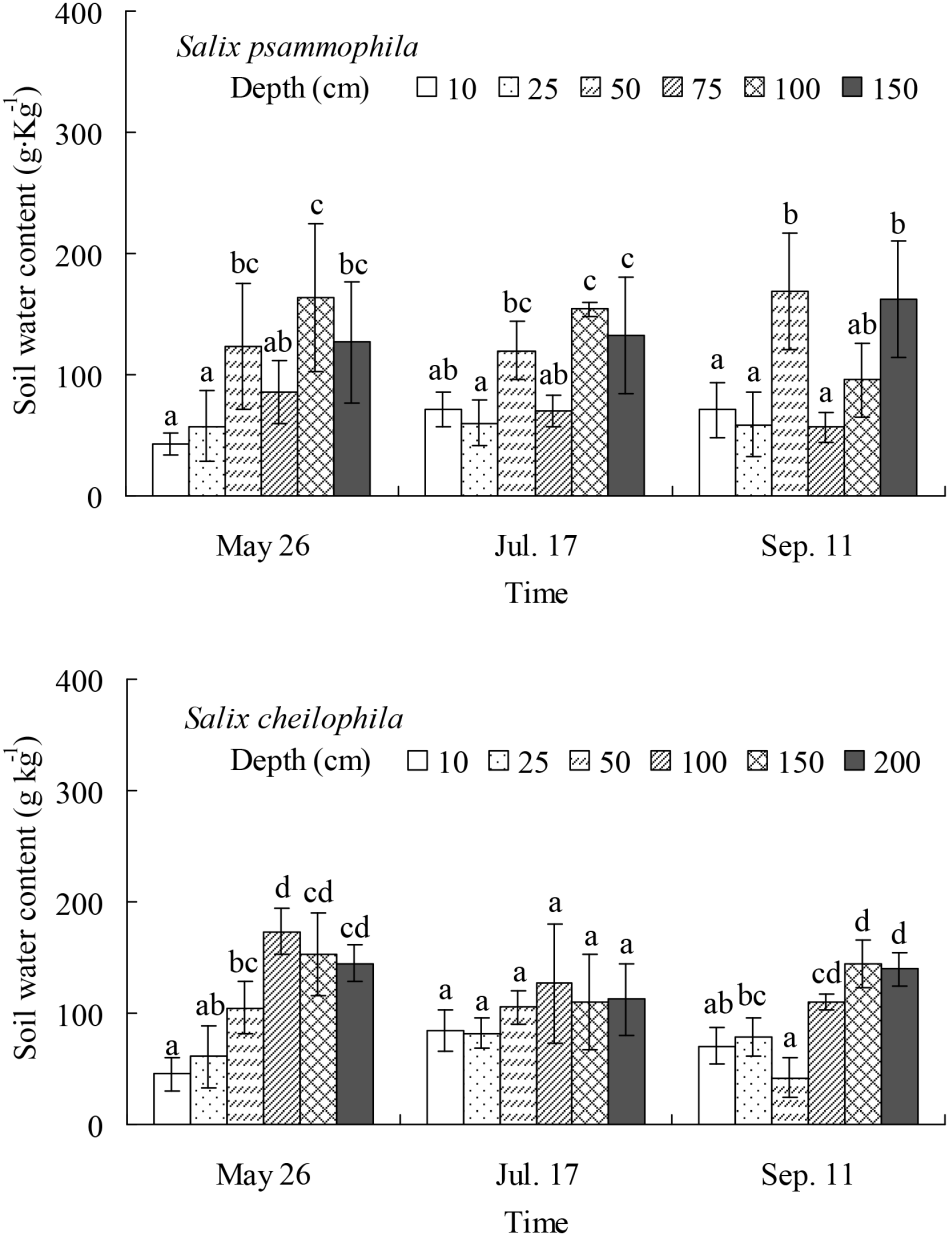
Soil water content at different soil depth for *Salix psammophila* and *S*. *cheilophila*. Different lower case letters indicate significant difference in soil water content at different depths and at different time according to Duncan’s test (*P* < 0.05).

Soil water content in *S*. *psammophila* shelterbelt was affected significantly by depth on May 26 (*P* < 0.01), July 17 (*P* < 0.001), and September 11 (*P* < 0.001) (Fig 2). Thus, on May 26 and July 17, the water content in deep soil (> 120 g kg^-1^, depth 50, 100, and 150 cm) was significantly higher than that in shallow soil (< 60 g kg^-1^, depth 10 and 25 cm on May 26; 59.97 g kg^-1^, depth 25 cm on July 17). On September 11, the water content (> 160 g kg^-1^) in middle soil (depth 50 cm) and the deepest soil (depth 150 cm) was significantly higher than that in shallow soil (< 70 g kg^-1^, depth 10 and 25 cm) and middle soil (< 60 g kg^-1^, depth 75 cm).

The soil water content in *S*. *cheilophila* shelterbelt was affected significantly by depth on May 26 (*P* < 0.001) and September 11 (*P* < 0.001), but it was not affected by depth on July 17 (*P* > 0.05) (Fig 2). On May 26, the water content in deep soil (> 100 g kg^-1^, depth 50–200 cm, > 100 g kg^-1^) was significantly higher than that in surface soil (45.51 g kg^-1^, depth 10 cm). On July 17, the soil water content was similar at different depths (83.92–126.48 g kg^-1^). On September 11, the water content in deep soil (> 100 g kg^-1^, depth 100–200 cm) was significantly higher than that in the surface soil (70.51 g kg^-1^, depth 10 cm) and middle soil (42.01 g kg^-1^, depth 50 cm).

### δD and δ^18^O Value of Xylem Water in the Two *Salix* Shrubs, Soil Water at Different Depths, Rain Water, and Ground Water

**Fig 3:**
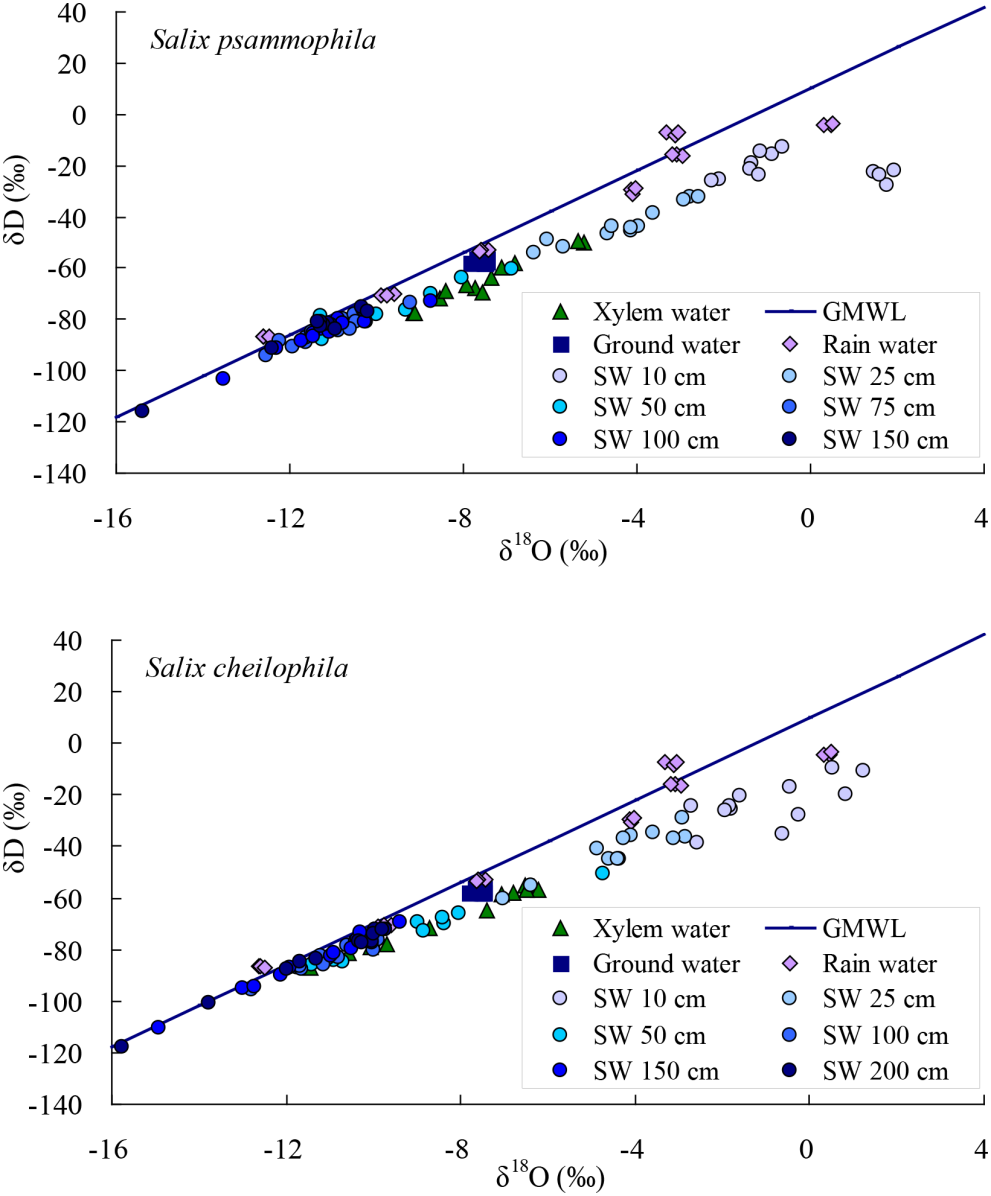
The value of δD and δ^18^O in xylem and soil water of *Salix psammophila* and *S*. *cheilophila*, ground water, rain water, and global meteoric water line (GMWL, δD = 8×δ^18^O+10) [11]. SW is soil water.

The δ^18^O value of xylem water in *S*. *psammophila* and *S*. *cheilophila* is located below the global meteoric water line (Fig 3), which indicates that the water source of the two shrubs was affected by isotope enrichment induced by evaporation. The δD and δ^18^O value of the ground water were closest to xylem water and soil water, indicating that the two shrubs used ground water and that soil water was recharged by the ground water. Some of the δD and δ^18^O value for rain water were closest to that of soil water, suggesting that soil water is recharged by the rain water.

**Fig 4:**
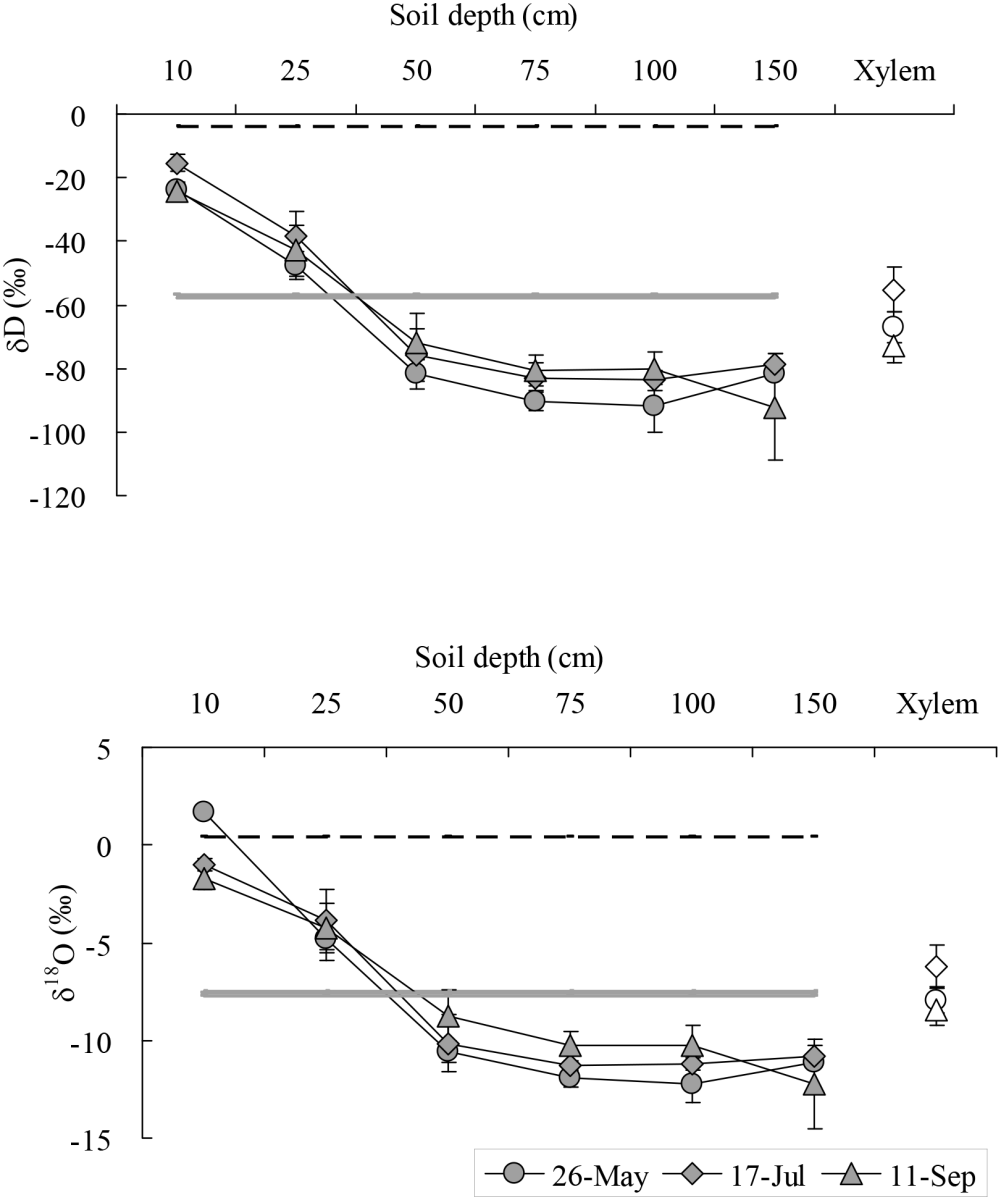
The value of δD and δ^18^O in xylem water of *Salix psammophila*, soil water, and ground water. Full line is ground water. Dotted line is the precipitation of 6.4 mm that occurred on July 8. Hollow symbols are xylem water. Solid symbols are soil water.

On May 26, the value of δD and δ^18^O in xylem water (−67.05‰, −7.94‰) of *S*. *psammophila* was closest to the value in ground water (−57.80‰, −7.50‰) and soil water at soil depths of 25 cm (−47.54‰, −4.78‰) and 50 cm (−81.84‰, −10.58‰) (Fig 4). On July 17, the value of δD and δ^18^O in xylem water (−55.23‰, −6.20‰) of *S*. *psammophila* was closest to the value in ground water and soil water at soil depths of 25 cm (−38.43‰, −3.85‰) and 50 cm (−75.75‰, −10.17‰). The value of δD and δ^18^O of soil water (−15.35‰, −1.02‰) at a soil depth of 10 cm was closest to that of rain water (−4.04‰, 0.43‰) on July 8 (6.4 mm). The rain water and ground water was accounted for 81.81% and 18.19% of its total source, respectively. On September 11, the value of δD and δ^18^O in xylem water (−72.69‰, −8.44‰) of *S*. *psammophila* was closest to that in ground water (−56.80‰, −7.71‰) and soil water at soil depth of 50–150 cm (−71.90‰ ~ −80.54‰, −8.79‰ ~ −10.29‰) (Fig 4).

**Fig 5:**
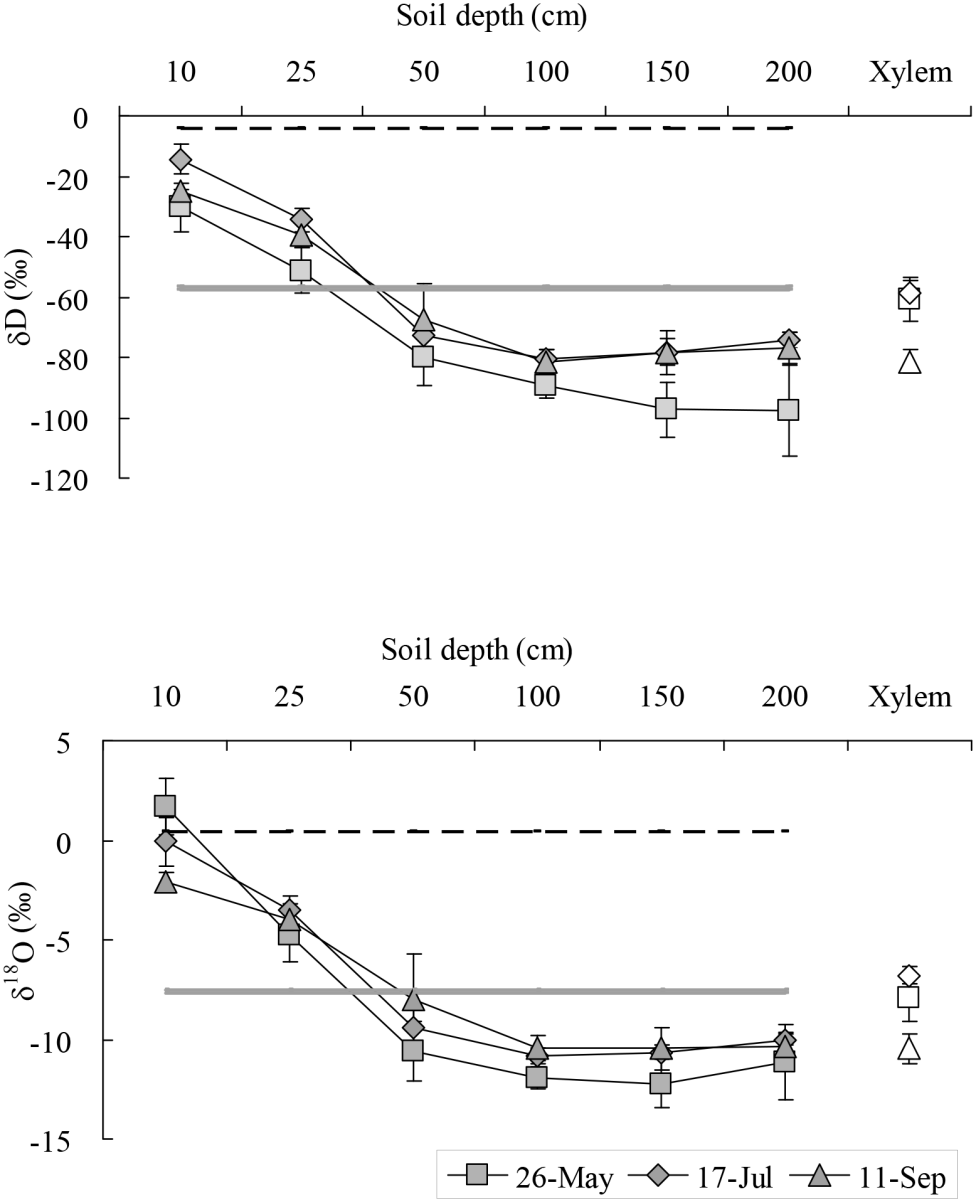
The ratio value of δD and δ^18^O in xylem water of *Salix cheilophila*, soil water, and ground water. Full line is ground water. Dotted line is the precipitation of 6.4 mm that occurred on July 8. Hollow symbols are xylem water. Solid symbols are soil water.

On May 26, the value of δD and δ^18^O in xylem water (−60.62‰, −7.08‰) of *S*. *cheilophila* was closest to that in ground water (−57.80‰, −7.50‰) and soil water at soil depths of 25 cm (−51.30‰, −5.60‰) and 50 cm (−80.21‰, −10.27‰) (Fig 5). On July 17, the value of δD and δ^18^O in xylem water (−58.62‰, −6.80‰) of *S*. *cheilophila* was closest to that in ground water (−56.70‰, −7.54‰). The value of δD and δ^18^O of soil water at a soil depth of 10 cm (−14.33‰, −0.08‰) was closest to that in rain water (−4.04‰, 0.43‰) on July 8 (6.4 mm). The rain water and ground water was accounted for 93.6%% and 6.4% of its total source, respectively. On September 11, the value of δD and δ^18^O in xylem water (−81.33‰, −10.45‰) of *S*. *cheilophila* was closest to that in ground water (−56.80‰, −7.71‰) and soil water at soil depths of 50–200 cm (−67.59‰ ~ −81.36‰, −8.00‰ ~ −10.47‰) (Fig 5).

### Water Use of the Two *Salix* Shrubs in Relation to Different Sources

The IsoSource analysis showed that *S*. *psammophila* used evenly soil water and ground water from different soil depths on May 26. On July 17, it absorbed primarily soil water at depths of 10 and 25 cm and ground water, accounting for 63.0% of its total water source. On September 11, this species utilized mainly soil water at a depth of 50–150 cm and ground water, accounting for 79.6% of its total water source. The use ratios of *S*. *psammophila* for ground water were 13.3%, 13.8% and 16.1% on May 26, July 17 and September 11, respectively (Table 1).

**Table 1:** Water use ratio (%) of *Salix psammophila* for different sources (n = 4, mean ± SD).

Water source	May 26	Jul. 17	Sep. 11
soil water at 10 cm depth	13.4 ± 5.2	26.2 ± 12.0	9.0 ± 6.3
soil water at 25 cm depth	18.2 ± 12.1	23.0 ± 16.9	11.3 ± 8.4
soil water at 50 cm depth	15.1 ± 12.1	9.9 ± 8.6	16.8 ± 14.6
soil water at 75 cm depth	12.9 ± 10.0	8.9 ± 7.7	16.1 ± 13.1
soil water at 100 cm depth	12.6 ± 9.7	8.9 ± 7.7	16.2 ± 13.2
soil water at 150 cm depth	14.8 ± 12.4	9.3 ± 0.8	14.4 ± 10.9
ground water	13.1 ± 8.9	13.8 ± 12.0	16.1 ± 13.7

The IsoSource analysis showed that *S*. *cheilophila* used mainly soil water at depths of 10 and 25 cm and ground water on May 26, accounting for 70.7% of its total water source. On July 17, this shrub used soil water and ground water evenly from different depths of soil profile. On September 11, it mainly used soil water at a depth of 100–200 cm, accounting for 81.8% of its total water source. The use ratios of *S*. *cheilophila* for ground water were 22.6%, 15.6% and 5.5% on May 26, July 17 and September 11, respectively (Table 2).

**Table 2:** Water use ratio (%) of *Salix cheilophila* for different sources (n = 4, mean ± SD).

Water source	May 26	Jul. 17	Sep. 11
soil water at 10 cm depth	16.7 ± 7.1	17.0 ± 8.8	2.2 ± 2.1
soil water at 25 cm depth	31.4 ± 20.2	18.8 ± 13.2	2.9 ± 2.7
soil water at 50 cm depth	9.2 ± 7.7	13.1 ± 11.2	7.6 ± 6.8
soil water at 100 cm depth	7.7 ± 6.4	11.5 ± 9.7	27.9 ± 19.9
soil water at 150 cm depth	6.2 ± 5.2	11.7 ± 9.9	28.0 ± 19.9
soil water at 200 cm depth	6.2 ± 5.2	12.3 ± 10.5	25.9 ± 19.9
ground water	22.6 ± 12.2	15.6 ± 13.6	5.5 ± 0.5

### Root Length and Mass Density of the Two *Salix* Shrubs

**Fig 6:**
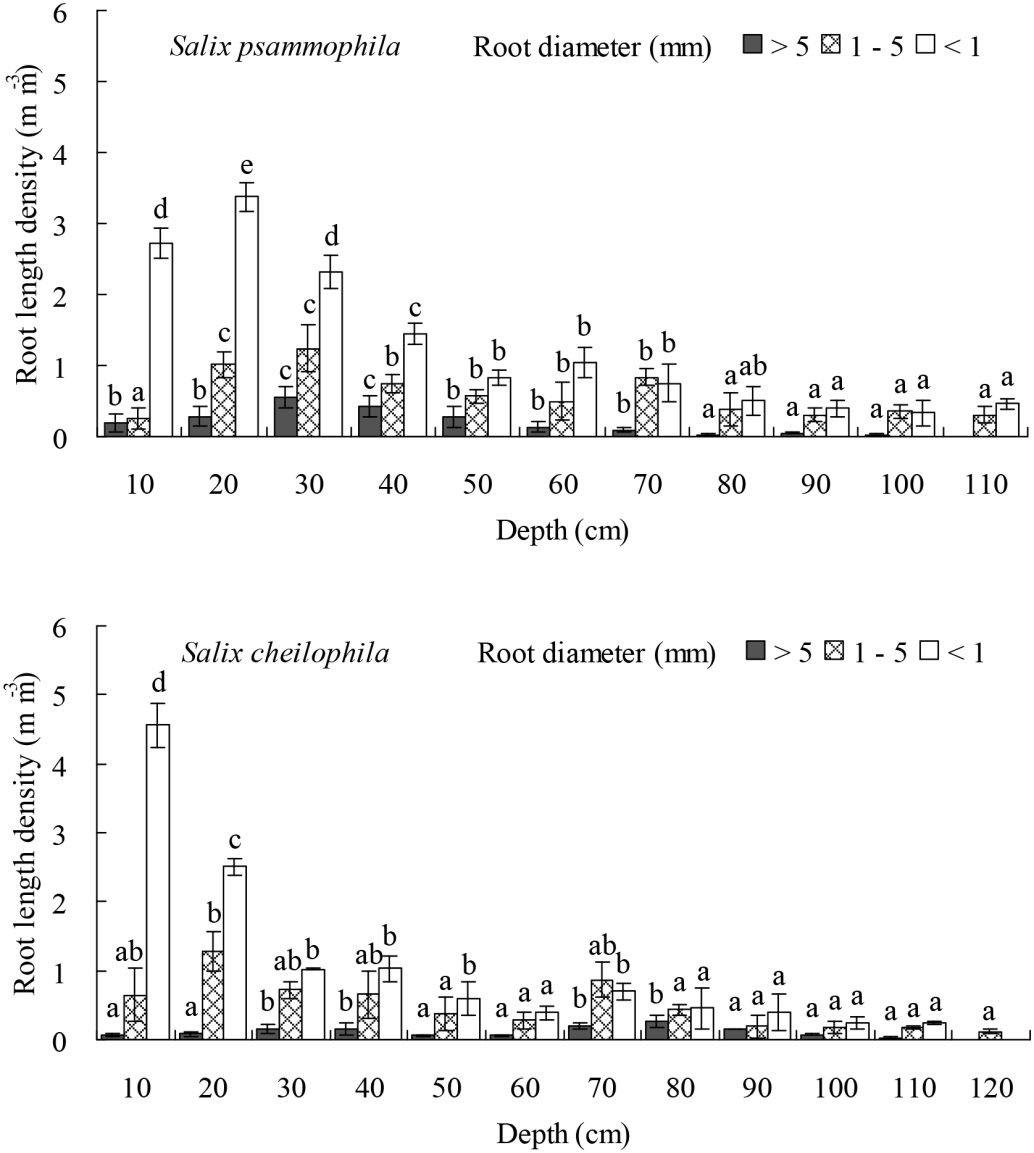
Root length density of *Salix psammophila* and *S*. *cheilophila* at different soil depth. Different lower case letters indicate significant difference in root length density at different soil depths according to Duncan’s test (*P* < 0.05).

The length density of prop roots (*P* < 0.05), medium roots (*P* < 0.05), and fine roots (*P* < 0.001) of *S*. *psammophila* and *S*. *cheilophila* changed significantly at different soil depths (Fig 6). Thus in *S*. *psammophila* stands, the length density of prop roots in the top 0–50 cm of soil profile accounted for 83.59% of its total length density. The length density of medium roots in the top 0–70 cm of soil profile accounted for 79.14% of its total length density. The length density of fine roots at a soil depth of 0–60 cm accounted for 82.63% of its total length density. In *S*. *cheilophila* stands, the length density of prop roots in the top 0–80 cm of soil profile accounted for 79.74% of its total length density. The length density of medium roots at a soil depth of 0–70 cm accounted for 81.46% of its total length density. The length density of fine roots in the top 0–50 cm layer of soil accounted for 80.05% of its total length density.

**Fig 7:**
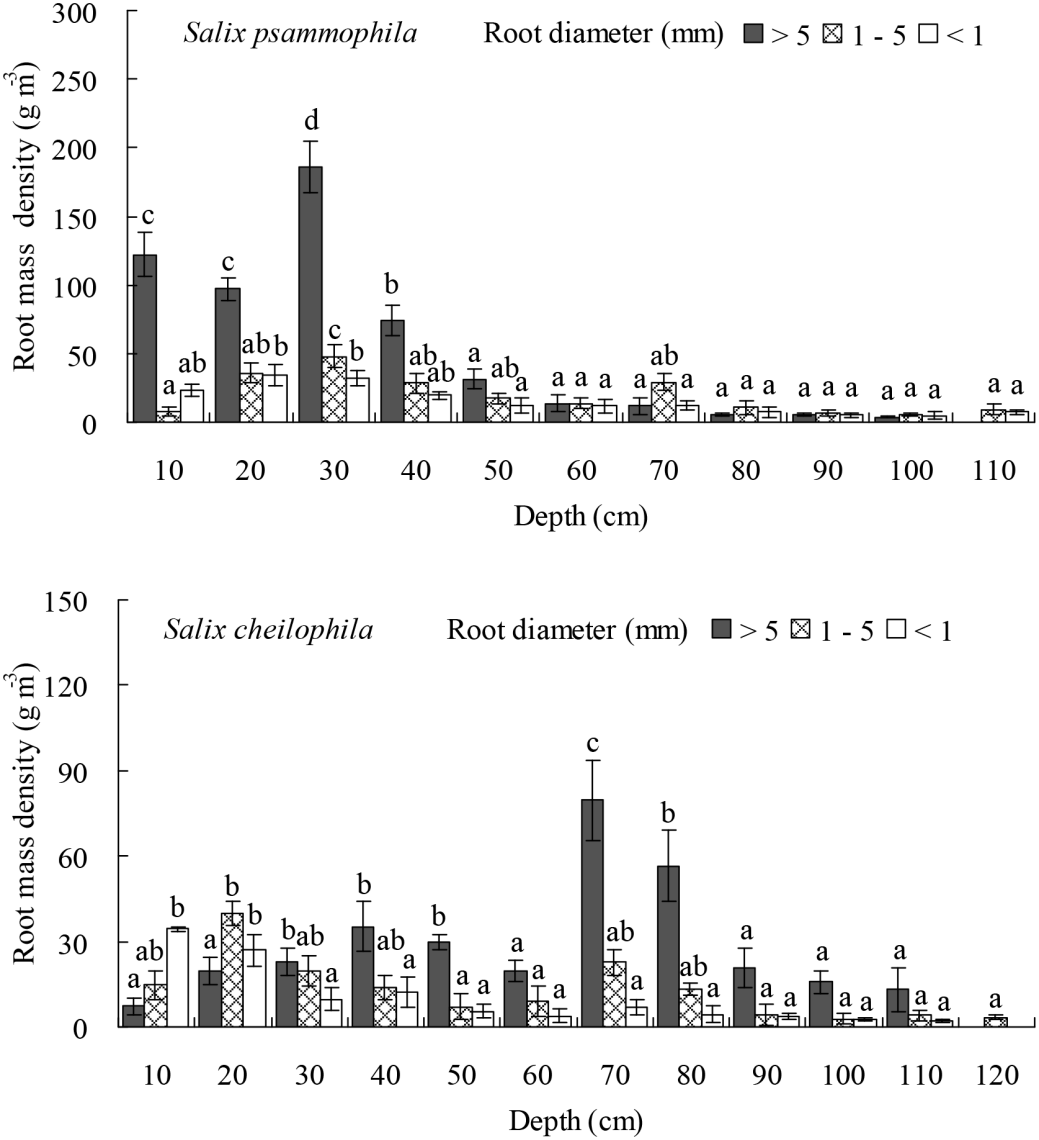
Root mass density of *Salix psammophila* and *S*. *cheilophila* at different soil depth. Different lower case letters indicate significant difference in root mass density at different soil depths according to Duncan’s test (*P* < 0.05).

Similarly, the mass density of prop roots (*P* < 0.05), medium roots (*P* < 0.001), and fine roots (*P* < 0.001) of both *S*. *psammophila* and *S*. *cheilophila* changed significantly at different depths (Fig 7). Thus in *S*. *psammophila* stands, the mass density of prop roots at a soil depth of 0–40 cm accounted for 87.03% of its total mass density. The mass density of medium roots in the top layer at 0–70 cm depth accounted for 84.68% of its total mass density. The mass density of fine roots in the soil layer at a depth of 0–70 cm accounted for 85.48% of its total mass density. In *S*. *cheilophila* stands, the mass density of prop roots at a depth of 0–80 cm accounted for 84.46% of its total mass density. The mass density of medium roots in the top 0–70 cm of soil profile accounted for 81.88% of its total length density. The mass density of fine roots at a soil depth of 0–50 cm accounted for 80.05% of its total mass density.

### Leaf δ^13^C Value and Water Use Efficiency of the Two *Salix* Shrubs

**Fig 8:**
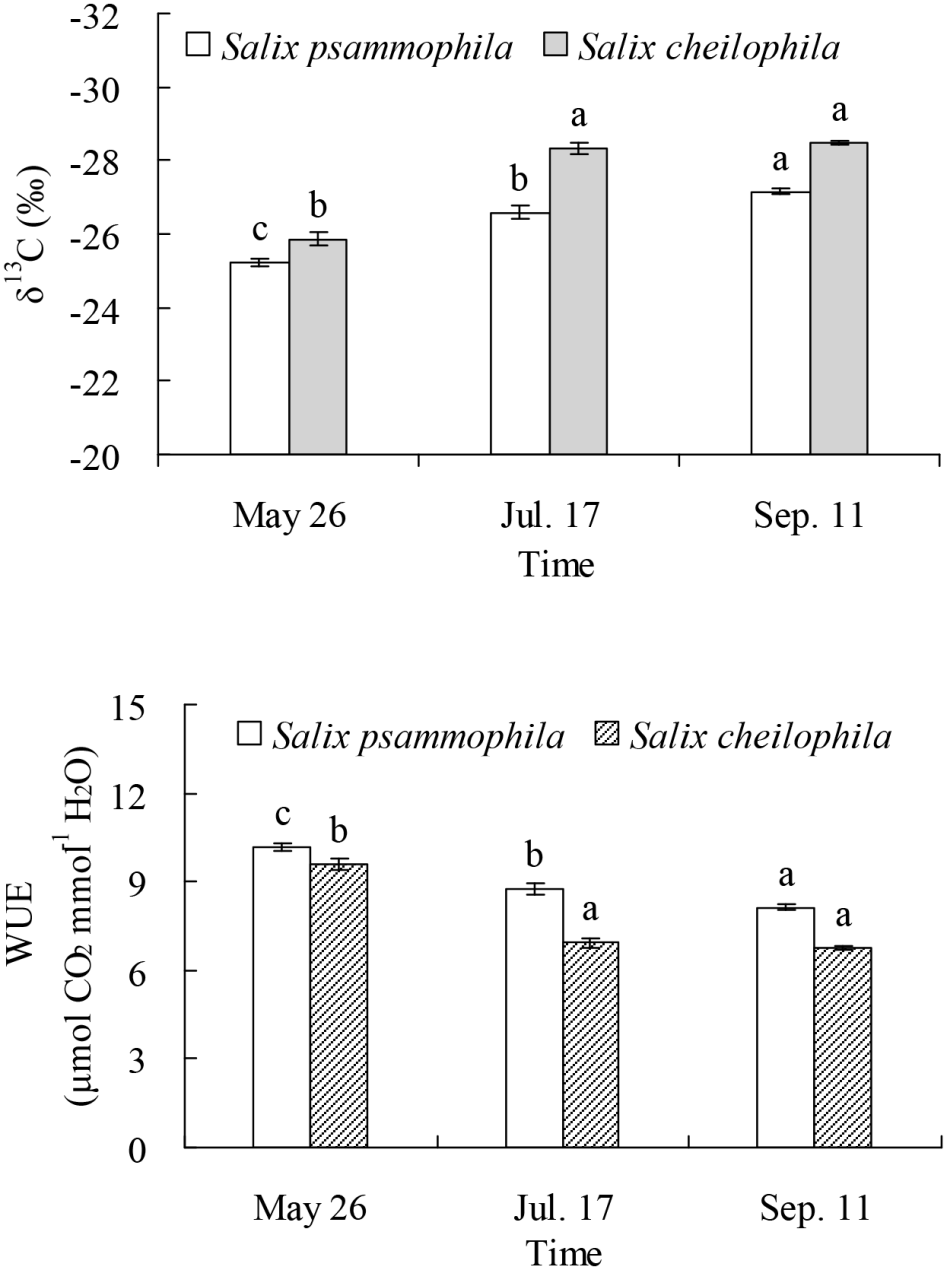
Leaf δ^13^C value and water use efficiency of *Salix psammophila* and *S*. *cheilophila*. Different lower case letters indicate significant difference in leaf δ^13^C value and water use efficiency in different months according to Duncan’s test (*P* < 0.05).

The leaf δ^13^C value of *S*. *psammophila* (*P* < 0.001) and *S*. *cheilophila* (*P* < 0.001) were significantly different in different months (Fig 8). The leaf δ^13^C value of *S*. *psammophila* dropped significantly from May 26 (−25.20‰) to July 17 (−26.57‰) and to September 11 (−27.16‰). The leaf δ^13^C value of *S*. *cheilophila* dropped significantly from May 26 (−25.85‰) to July 17 (−28.32‰) or September 11 (−28.49‰).

Water use efficiency of *S*. *psammophila* (*P* < 0.001) and *S*. *cheilophila* (*P* < 0.001) were significantly different in different months (Fig 8). Water use efficiency of *S*. *psammophila* dropped significantly from May 26 (10.16 μmol CO_2_ mmol^-1^ H_2_O) to July 17 (8.74 μmol CO_2_ mmol^-1^ H_2_O) and to September 11 (8.12 μmol CO_2_ mmol^-1^ H_2_O). Water use efficiency of *S*. *cheilophila* dropped significantly from May 26 (9.49 μmol CO_2_ mmol^-1^ H_2_O) to July 17 (6.93 μmol CO_2_ mmol^-1^ H_2_O) or September 11 (6.75 μmol CO_2_ mmol^-1^ H_2_O).

## Discussion

### Seasonal Dynamics of the Main Water Source of the Two *Salix* Shrubs

The two *Salix* shrubs growing on interdune of the alpine sandy land used soil water at different depths depending on their availability in different seasons. Moreover, the reliance on the ground water was different between the two *Salix* shrubs. *Salix psammophila* used evenly soil water at a soil depth of 10–150 cm and ground water in spring, shallow soil water recharged by rain at a soil depth of 10–25 cm and ground water in summer, and medium and deep soil water and ground water at a soil depth of 50–150 cm in fall (Fig 4, Table 1). However, *S*. *cheilophila* used shallow soil water recharged by rain at a soil depth of 10–25 cm and ground water in spring, soil water at a soil depth of 10–200 cm and ground water in summer, and deep soil water recharged by ground water at a soil depth of 100–200 cm in fall (Fig 5, Table 2). Therefore, ground water and deep soil water recharged by ground water were the main long-term stable water sources for the two *Salix* shrubs. Moreover, *S*. *psammophila* and *S*. *cheilophila* also used shallow soil water recharged by rain in spring and summer, respectively (Fig 1). Similarly, *Caragana intermedia* growing on sand dunes always utilized shallow soil water at a soil depth of 0–50 cm during the growing season [13]. Thus, desert shrubs use different water sources depending on the availability of the resources during the growing season, including soil water recharged by rain or ground water.

The resource-dependent water use strategy of the two *Salix* shrubs in alpine sandy land of Tibetan Plateau is similar to that reported in other trees and shrubs in arid and semi-arid regions. Some trees and shrubs only used soil water recharged by rain. *Chrysothamnus greenei* absorbed only soil water recharged by rain in the San Luis Valley, Colorado [37]. *Pinus sylvestris* var. *mongolica* primarily used soil water at a soil depth of 20–60 cm both at the top of fixed sand dunes and in the interdune lowland in Horqin Sandy Land [26]. Other trees and shrubs used both of deep soil water and ground water. *Salix matsudana* and *Sabina vulgaris* mainly used deep soil water and ground water in Mu Us Sandy Land [24]. *Nitraria tangutorum* in the Golmud of Qaidam Basin mainly utilized soil water at a soil depth of 50–100 cm and ground water from June to September [38]. Some trees and shrubs used different water source in the growing season. *Juniperus osteosperma* absorbed shallow soil water in early spring and gradually increased its dependency on deep soil water with increase in soil drought in Utah [16]. During the high abundance of upper soil water in early spring, *Haloxylon ammodendron* primarily utilized shallow soil water on sand dunes of Gurbantonggut Desert, whereas in summer, when the upper soil water was depleted, this species mainly utilized ground water [21]. In Qaidam Basin, *N*. *tangutorum* and *Tamarix ramosissima* used soil water at a soil depth of 50–70 cm, whereas *Ephedra sinica* and *Calligonum mongolicum* utilized evenly soil water at a soil depth of 0–90 cm. Moreover, these four shrubs increased the use of ground water in the late growing season [39]. In addition, some plant species used different water source in different years, depending on annual precipitation. *Sarcobatus vermiculatus* and *Chrysothamnus nauseosus* used soil water recharged by rain in a wet year but deep soil water and ground water in a dry year in the San Luis Valley, Colorado [37]. *Pinus sylvestris* var. *mongolica* only absorbed soil water during higher precipitation year but used soil water and relied on ground water during lower precipitation year in Horqin Sandy Land [27]. However, few trees always used ground water. *Ulmus pumila* always utilized stable ground water in Hunshandake Sandy Land [14]. Therefore, ground water is an important water source for trees and shrubs in arid and semi-arid regions, especially during the dry period. Global climate change may result in decreased mean precipitation and extreme increase in drought in arid regions [8]. Under such conditions, plant species that use stable ground water or deep soil water may be better adapted than those that only use shallow soil water during the period of drought.

### Root Distribution Pattern of the Two *Salix* Shrubs

The maximal depths reached by the root system of *S*. *psammophila* and *S*. *cheilophila* were 1.1 m and 1.2 m, respectively, on interdune of the alpine sandy land. The length and biomass of prop roots was mainly distributed in the top 0–50 cm and 0–80 cm of the soil for *S*. *psammophila* and *S*. *cheilophila*, respectively. The length and biomass of medium roots was mainly distributed in the top soil layer at a depth of 0–70 cm for both species. The length of fine roots was concentrated at a soil depth of 0–70 cm for *S*. *psammophila* and 0–90 cm for *S*. *cheilophila* (Fig 6). However, the root biomass was mainly distributed at a depth of 0–70 cm and 0–40 cm, respectively (Fig 7). Therefore, root distribution of the two *Salix* shrubs was dimorphic, allowing them to use soil water recharged by rain or ground water. Similarly, the root system of *S*. *vulgaris* is distributed from soil surface to soil layers above the ground water table (1.2 m) in Mu Us Sandy Land, and therefore, this species is able to absorb soil water at many depths as well as utilize the ground water [24]. In the present study, prop and fine root distribution was different in the two *Salix* shrubs. In general, the root mass of *S*. *psammophila* was higher than that of *S*. *cheilophila*. The prop roots of *S*. *cheilophila* reached deeper soil layers compared to *S*. *psammophila*, whereas the biomass of the fine roots was greater in *S*. *psammophila*, although the fine roots were longer, reaching deeper soil layers in *S*. *cheilophila*. Therefore, *S*. *psammophila* utilizes deeper soil water efficiently by its higher biomass of fine roots, whereas *S*. *cheilophila* developed longer roots but invested less in their biomass to access water at greater depths.

Water source of different shrubs is closely related to their root distribution pattern in desert ecosystems. Deep-rooted shrubs use stable resources of deep soil water or ground water. For example, *Ericameria nauseosa* depended on ground water and *Sarcobatus vermiculatus* used ground water during dry periods but absorbed deep soil water after large rainfalls in the San Luis Valley of Colorado [23]. *Tamarix chinensis*, *Alhagi sparsifolia*, *Elaeagnus angustifolia*, and *Nitraria sphaerocarpa* absorbed soil water at depths greater than 80 cm in Dunhuang [40]. Shallow-rooted shrubs use shallow soil water. For example, *Senecio filaginoides* and *Mulinum spinosum* in the Patagonian steppe absorbed soil water from soil layers at a soil depth of 10 cm [19]. Some shrubs with a dimorphic root system are able to use both shallow and deep soil waters [13].

### Seasonal Dynamics of Water Use Efficiency of the Two *Salix* Shrubs

The leaf δ^13^C value in the two *Salix* shrubs growing on interdune of Gonghe Basin indicated the presence of seasonal dynamics of long-term water use efficiency, which were higher in spring than in summer and fall (Fig 8). The soil water content at soil surface was lower than that in deeper soil layers in spring (Fig 2) because of low precipitation (Fig 1). The data suggest that both *Salix* shrubs increase water use efficiency to adapt to drought. Similarly, the long-term water use efficiency of *C*. *intermedia* was highest in spring on sand dune of Gonghe Basin [13]. However, the water use efficiency of *P*. *sylvestris* var. *mongolica* was constant for two years in Keerqin Sandy Land, indicating that it did not suffer severely from water stress [27].

The results of our study indicate the intra-specific difference in water use efficiency between the two *Salix* shrubs growing on interdune of the Gonghe Basin. The water use efficiency of *S*. *psammophila* was higher than that in *S*. *cheilophila* (Fig 8). Difference in water use efficiency of different plant species is related to their life form. *Salix psammophila* is a psammophyte whereas *S*. *cheilophila* is hygro-mesophyte. Similarly, the evergreen *S*. *vulgaris* had higher water use efficiency than *S*. *matsudana* and *A*. *ordosica* in Mu Us Sandy Land [24]. The long-term water use efficiency of the evergreen *Ammopiptanthus mongolicus*, *N*. *tangutorum*, and *C*. *korshinskii* was higher than that of *A*. *ordosica* in Ulanbuh Desert [41]. In arid and semi-arid regions, shrubs with higher water use efficiency may be better adapted to extreme drought caused by global climate change.

## Conclusions

In alpine sandy land of the Tibetan Plateau, *S*. *psammophila* and *S*. *cheilophila* growing on the interdune used soil water at different soil depths or ground water, depending on water availability in the growing season. *Salix psammophila* continuously used ground water in the growing season and relied on shallow soil water in summer. *Salix cheilophila* relied on shallow and deep soil water in spring and fall, respectively, and used ground water in spring and summer. The two *Salix* shrubs had dimorphic root system, which is coincident with their water use strategy. *Salix psammophila* and *S*. *cheilophila* are able to access deeper soil water efficiently thanks to higher biomass and greater root length of fine roots, respectively. The long-term water use efficiency of the two *Salix* shrubs increased under drought conditions in spring. The long-term water use efficiency of *S*. *psammophila* was higher than that of *S*. *cheilophila*, which might be due to its better adaptation to the semi-arid climate in alpine sandy land. These findings will contribute to development of vegetative rehabilitation and ecological restoration of arid and semi-arid regions. It is suggested that large area plantation of two *Salix* shrubs should be limited to avoid excessive consumption of ground water in alpine sandy land of Tibetan Plateau.

## Supporting Information

S1 File All data of Figs 1–8 are available in the file. (XLS)

## References

[1] Zhang D, Gao S, Shi M, Hasi E, Yan P, Lu R (2009) Land Desertification and Its Control in Plateau of Qinghai. Beijing: Science Press. (in Chinese).

[2] Harris RB (2010) Rangeland degradation on the Qinghai-Tibetan Plateau: A review of the evidence of its magnitude and causes. J Arid Environ 74:1–12.

[3] Feng Y, Lu Q, Wu B, Liu H, Wang X, Che T (2011) Land-use dynamics of alpine-cold desertified area in the Qinghai-Tibetan Plateau in the last 30 years: A case study in Guinan County, Qinghai Province, China. Int J Sust Dev World Ecol 18: 357–365.

[4] Zhu Y, Li H, Zhao S, Jia Z, Yu Y, Li Q (2014) Improvement effect on microclimate in different types of shelterbelt in the Gonghe Basin of Tibet Plateau. J Des Res 34: 814–818. (in Chinese with English abstract).

[5] Ehleringer JR (1993) Carbon and water relations in desert plants: an isotopic perspective In: Ehleringer JR, Hall AE, Farquhar GD. Ed. Stable Isotope and Plant Carbon-Water Relations. San Diego: Academic Press pp. 155–172.

[6] Schwinning S, Starr BI, Ehleringer JR (2003) Dominant cold desert plants do not partition warm season precipitation by event size. Oecologia 136: 252–260. 12695904 10.1007/s00442-003-1255-y

[7] Hou Y, Zhou G, Xu Z, Liu T, Zhang X (2013) Interactive effects of warming and increased precipitation on community structure and composition in an annual forb dominated desert steppe. PLoS ONE 8: e70114 10.1371/journal.pone.0070114 23894600 PMC3716769

[8] IPCC (2013) Climate Change 2013 The Physical Science Basis. New York: Cambridge University Press.

[9] Ogle K, Reynolds JF (2004) Plant responses to precipitation in desert ecosystems: integrating functional types, pulses, thresholds, and delays. Oecologia 141: 282–294. 15007725 10.1007/s00442-004-1507-5

[10] Xia J, Wan S (2012) The effects of warming-shifted plant phenology on ecosystem carbon exchange are regulated by precipitation in a semi-arid grassland. PLoS ONE 7: e32088 10.1371/journal.pone.0032088 22359660 PMC3281109

[11] Lin G (2013) Stable Isotope Ecology. Beijing: Higher Education Press. (in Chinese).

[12] Dawson TE, Mambelli S, Plamboeck AH, Templer PH, Tu KP (2002) Stable isotopes in plant ecology, Annu Rev Ecol Syst 33: 507–559.

[13] Jia Z, Zhu Y, Liu L (2012) Different water use strategies of juvenile and adult *Caragana intermedia* plantations in the Gonghe Basin, Tibet Plateau. PLoS ONE 7: e45902 10.1371/journal.pone.0045902 23029303 PMC3448693

[14] Su H, Li Y, Liu W, Xu H, Sun OJ (2014) Changes in water use with growth in *Ulmus pumila* in semiarid sandy land of northern China. Trees 28: 41–52.

[15] Cheng X, An S, Li B, Chen J, Lin G, Liu Y, et al (2006) Summer rain pulse size and rainwater uptake by three dominant desert plants in a desertified grassland ecosystem in northwestern China. Plant Ecol 184: 1–12.

[16] West AG, Hultine KR, Burtch KG, Ehleringer JR (2007) Seasonal variation in moisture use in a piňon-juniper woodland. Oecologia 153: 787–798. 17576601 10.1007/s00442-007-0777-0

[17] McCole AA, Stern LA (2007) Seasonal water use pattern of Juniperus ashei on the Edwards Plateau, Texas, based on stable isotope in water. J Hydrol 342: 238–248.

[18] Li S-G, Romero-Saltos H, Tsujimura M, Sugimoto A, Sasaki L, Davaa G, et al (2007) Plant water sources in the cold semiarid ecosystem of the upper Kherlen River catchment in Mongolia: A stable isotope approach. J Hydrol 333: 109–117.

[19] Kowaljow E, Fernández RJ (2011) Different utilization of a shallow-water pulse by six shrub species in the Patagonian steppe. J Arid Environ 75: 211–214.

[20] Zhu Y, Jia Z, Yang X (2011) Resource-dependent water use strategy of two desert shrubs on interdune, Northwest China. J Food, Agri & Environ 9(3&4): 832–835.

[21] Dai Y, Zheng X-J, Tang L-S, Li Y (2015) Stable oxygen isotopes reveal distinct water use patterns of two *Haloxylon* species in the Gurbantonggut Desert. Plant Soil 389: 73–87.

[22] Yang H, Auerswald K, Bai Y, Han X (2011) Complementarity in water sources among dominant species in typical steppe ecosystems of Inner Mongolia, China. Plant Soil 340: 141–155.

[23] Kray JA, Cooper DJ, Sanderson JS (2012) Groundwater use by native plants in response to changes in precipitation in an intermountain basin. J Arid Environ 83: 25–34.

[24] Ohte N, Koba K, Yoshikawa K, Sugimoto A, Matsuo N, Kabeya N, et al (2003) Water utilization of trees in semiarid desert of Inner Mongolia, China. Ecol App 13: 337–351.

[25] Pataki DE, Bush SE, Gardner P, Solomon DK, Ehleringer JR (2005) Ecohydrology in a Colorado River riparian forest: Implications for the decline of *Populus fremontii*. Ecol App 15: 1009–1018.

[26] Wei YF, Fang J, Liu S, Zhao XY, Li SG (2013) Stable isotope observation of water use source of *Pinus sylvestris* var. *mongolica* in Horqin Sandy Land, China. Trees 27: 1249–1260.

[27] Song L, Zhu J, Li M, Yu Z (2014) Water utilization of *Pinus sylvestris* var. *mongolica* in a sparse wood grassland in the semiarid sandy region of Northeast China. Trees 28: 971–982.

[28] Liu S, Chen Y, Chen Y, Friedman JM, Hati JHA, Fang G (2015) Use of ^2^H and ^18^O stable isotopes to investigate water sources for different ages of *Populus euphratica* along the lower Heihe River. Ecol Res 30: 581–587.

[29] Cheng X, Huang M, Shao M, Warrington DN (2009) A comparison of fine root distribution and water consumption of mature *Caragana korshinkii* Kom grown in two soils in a semiarid region, China. Plant Soil 315: 149–161.

[30] Zeng FJ, Lu Y, Liu B, Guo HF, Zeng J, Zhang LG, et al (2013) One-year-old seedling biomass distribution and root architecture characteristics differed between two desert plants: *Tamarix ramosissima* and *Alhagi sparsifolia*. Arid Land Res Man 27: 298–302.

[31] Xu G, Li Y, Xu H (2011) Seasonal variation in plant hydraulic traits of two co-occurring desert shrubs, *Tamarix ramosissima* and *Haloxylon ammodendron*, with different rooting patterns. Ecol Res 26: 1071–1080.

[32] Zhu Y, Jia Z (2011) Soil water utilization characteristics of *Haloxylon ammodendron* plantation with different age during summer. Acta Ecologica Sinica 31: 341–346.

[33] Liu SW (1997) Flora Qinghaiica. Volume 1 Xining: Qinghai Pepole’s Publishing House pp. 117–118. (in Chinese).

[34] Ma YQ (1985) Flora Intramongolica. Volume 1 Huhhot: Inner Mongolia People Press pp. 53–54. (in Chinese).

[35] Farquhar GD, Hubick KT, Condon AG, Richards RA (1989) Carbon isotope fractionation and plant water-use Efficiency In Rundel PW, Ehleringer JR, Nagy KA (eds.) Stable Isotopes in Ecological Research. New York: Springer-Verlag pp. 21–40.

[36] Phillips DL, Gregg JW (2003) Source partitioning using stable isotopes: coping with too many sources. Oecologia 136: 261–269. 12759813 10.1007/s00442-003-1218-3

[37] Chimner RA, Cooper DJ (2004) Using stable oxygen isotopes to qualify the water source used for transpiration by native shrubs in San Luis Valley, Colorado U.S.A. Plant Soil 206: 225–236.

[38] Gong G, Chen H, Duan D (2011) Comparison of the methods using stable hydrogen and oxygen isotope to distinguish the wate*r source of Nitraria tangutorum*. Acta Ecologica Sinica 31: 7533–7541. (in Chinese with English abstract).

[39] Xing X, Chen H, Zhu J, Chen T (2014) Water sources of five dominant desert species in Nuomuhong area of Qaidam Basin. Acta Ecologica Sinica 34: 6277–6286. (in Chinese with English abstract).

[40] Cui YQ, Ma JY, Sun W, Sun JH, Duan ZH (2015) A preliminary study of water use strategy of desert plants in Dunhuang, China. J Arid Land 7: 73–81.

[41] Zhu Y, Jia Z, Lu Q, Hao Y, Zhang J, Li L, et al (2010) Water use strategy of five shrubs in Ulanbuh Desert. Scientia Silvae Sinicae 46(4): 15–21. (in Chinese with English abstract).

